# A Delusion and a Snare

**Published:** 1886-05

**Authors:** 


A DELUSION AND A SNARE. FALSE HOP(E)S.

"The new alkaloid recently announced as the active constituent of hops, and named by its discoverer "hopein," turns out to be morphine flavored with hops."—*American Lancet.*

So there is no use in hop(e)in'.

